# The Effects of Imprinting and Repeated Seasonal Influenza Vaccination on Adaptive Immunity after Influenza Vaccination

**DOI:** 10.3390/vaccines8040663

**Published:** 2020-11-07

**Authors:** Amy C. Sherman, Lilin Lai, Mary Bower, Muktha S. Natrajan, Christopher Huerta, Vinit Karmali, Jennifer Kleinhenz, Yongxian Xu, Nadine Rouphael, Mark J. Mulligan

**Affiliations:** 1 The Hope Clinic of Emory Vaccine Center, Emory University, Atlanta, GA 30030, USA; lilin.lai@nyu.langone.org (L.L.); mbbower@emory.edu (M.B.); muktha.natrajan@emory.edu (M.S.N.); christopher.m.huerta@emory.edu (C.H.); vkarmal@emory.edu (V.K.); jennifer.m.kleinhenz@emory.edu (J.K.); y.xu@emory.edu (Y.X.); nroupha@emory.edu (N.R.); mark.mulligan@nyulangone.org (M.J.M.); 2 Division of Infectious Diseases, Brigham and Women’s Hospital, Boston, MA 02115, USA; 3 Division of Infectious Diseases and Immunology, NYU Langone Vaccine Center, New York City, NY 10016, USA

**Keywords:** influenza, imprinting, birth cohort, repeated vaccination, adaptive immunology

## Abstract

(1) Background: The influenza virus continues to cause significant annual morbidity and mortality. The overall efficacy of seasonal influenza vaccination is suboptimal, which is partly due to host immune factors. The effects of imprinting and repeated seasonal influenza vaccination were investigated to assess for immune factors and mechanisms that impact influenza vaccine responses. (2) Methods: Twenty participants were enrolled into a prospective pilot study based on birth cohort and seasonal influenza immunization history. Immunologic parameters were assessed over a six-month period after the seasonal influenza vaccine was administered. (3) Results: There was no significant imprinting effect, as measured by hemagglutination inhibition (HAI) fold change, HAI geometric mean titer (GMT) for Day 29 or Day 180 post-vaccination and antigen- specific antibody-secreting cells (ASC) for Day 8 post-vaccination. Individuals who had minimal prior seasonal influenza vaccination had a higher magnitude ASC response and a higher HAI fold change post-vaccination than individuals who were repeatedly vaccinated. (4) Conclusions: Repeated seasonal influenza vaccination resulted in a decreased fold change of the immune response, although individuals in this cohort tended to have high HAI titers at baseline that persisted after vaccination. Imprinting effects were not observed in this cohort. These host immune factors should be considered in the development of universal influenza vaccines. ClinicalTrials.gov Identifier: NCT03686514.

## 1. Introduction

Seasonal influenza outbreaks continue to cause substantial disease burden, with an estimated 3–5 million cases of severe illness, and 290,000 to 650,000 deaths worldwide each year [1]. In the United States, influenza has resulted in 9–45 million illnesses, with 12,000–61,000 deaths annually since 2010 [2]. There is an urgent need to better understand the immunologic responses to current licensed influenza vaccines in order to develop a more effective vaccine that does not rely on annual strain-matched boosters, provides broad protection, and is durable, i.e., a universal influenza vaccine. Two factors that may affect the immune response to influenza vaccination include imprinting and repeated annual vaccination. 

In 1960, Francis described the “doctrine of original antigenic sin (OAS) [3].” He observed that the antibodies produced by a child to the first influenza A subtype infection continued to dominate throughout his or her life and governed the immune response to all subsequent influenza exposures. Evidence for OAS has been described since, and highlights the importance of pre-existing B cell memory on the influenza specific immune response [4,5]. Gostic et al. demonstrated that an individual has a lifelong “imprint” from his or her first influenza A virus (IAV) exposure, which then reduces the risk of severe disease not only against subsequent exposures to the same strain but also against novel strains of IAV within the same phylogenetic group (e.g., hemagglutinin (HA) group 1 includes subtypes H1, H2, and avian H5, while HA group 2 includes seasonal H3 and avian H7). Amino acid homology for the conserved HA stem region is significantly higher within groups, as opposed to between groups [6]. Further evidence of imprinting was described by Arevalo et al., who used mathematical modeling from data of influenza vaccine effectiveness studies to demonstrate that primary influenza infection reduced the risk of medically attended infection with that particular subtype throughout life, with a stronger effect seen for H1N1 than for H3N2 [7]. Birth year-specific differences based upon childhood imprinting have also been compared to differences in the evolutionary rate of H1N1 versus H3N2, with the discovery that the imprinting effects were the driving factor in infection risk differences from seasonal influenza [8]. In a large Vietnamese cohort of unvaccinated individuals, the highest serologic titers were for influenza viruses that had circulated when the individuals were around six years of age, which was likely the time frame of their first infections, but may have missed detection of earlier imprinting [9]. Thus, birth-year cohorts can be defined based on an individual’s initial childhood exposure to H1N1, H3N2, or another IAV sub-type.

Other studies have also alluded to the significance of imprinting in terms of epidemiological patterns seen during influenza outbreaks. For example, the 2009 A(H1N1) pandemic was unusual: mortality was lower in the elderly when compared to usual influenza outbreak trends, and higher mortality was observed in young and middle-aged adults [10]. Protection against the pandemic IAV strain has been well described in older cohorts due to prior exposure to antigenically related strains, either from natural infection or from those immunized against the 1976 New Jersey A(H1N1) virus, a finding that supports the imprinting phenomenon [11,12,13]. A retrospective study by Flannery et al. examined data from the Flu VE Network study and demonstrated that patients’ initial infections with specific A(H1N1) virus clades influenced vaccine efficacy after exposure to A(H1N1). Birth cohorts were based upon estimated immunologic priming with A(H1N1) viral clades circulating from 1918–1957 and from 1977–2015 [14]. Similarly, vaccine effectiveness (VE) for the 2015–2016 influenza season was analyzed, with the finding that VE against A(H1N1) pdm09 was decreased in individuals born between 1957 and 1976, who had likely imprinted upon other subtypes [15]. Gagnon et al. reported a potentially more complex phenomenon in individuals who were born during heterosubtypic pandemics. Individuals born during the H2N2 pandemic had higher mortality in 2009 and 2013–2014 for pandemic H1N1 [16], suggesting that this cohort may actually have a greater risk for mortality for subsequent heterosubtypic pandemics. 

The effect of repeated annual influenza vaccination is another factor that may influence the host immune response to influenza. Hoskins first questioned the effects of repeated influenza vaccination in 1979, when he observed that children who had received consecutive seasons of influenza immunization actually appeared to be at higher risk of contracting influenza disease [17]. Epidemiological studies have evaluated the clinical effectiveness of seasonal influenza immunization among persons who had repeated annual vaccinations during different seasons, with several analyses showing poor vaccine efficacy among this group [18,19,20,21,22,23]. However, other analyses demonstrated continued protection with repeated annual influenza vaccination [24,25], or determined that the evidence is inconclusive to advise against repeated influenza vaccination [26,27]. Thus, there are discrepant results and the underlying immune characteristics affecting those who have been repeatedly vaccinated, as compared with persons who are minimally vaccinated or unvaccinated, are not well described. 

This pilot study is important to identify the immune responses that occur due to both imprinting (at the HA phylogenetic group level) and repeated influenza vaccination, and to determine which of these factors is most influential in influenza-specific immune response to seasonal influenza vaccination. 

## 2. Materials and Methods

### 2.1. Study Design

A prospective pilot study, with two groups of 10 participants each, was conducted at the Hope Clinic of the Emory Vaccine Center during the 2018–2019 influenza season. The H3N2 group (*n* = 10) consisted of healthy participants born between 1968–1977, when H3N2 was the primary IAV circulating in the US. The H1N1 group (*n* = 10) consisted of participants born between 1948 and 1957, when H1N1 was the primary IAV circulating in the US. Each group was further stratified by participants who received the seasonal influenza vaccine two times or less in the past five seasons, and participants who received the influenza vaccine three or more times in the past five seasons. Once informed consent was obtained, study procedures were performed and subjects were followed for a period of 6 months. Baseline phlebotomy was obtained on Day 1, followed by intramuscular administration of the FDA approved 2018–2019 quadrivalent influenza vaccine (one 0.5 mL dose to the deltoid muscle lot number 75TA2, Fluarix, GSK, Brentford, UK) between October 2018 and January 2019. The components of the vaccine are listed (Table 1). Subsequent study visits occurred on Days 3, 8, 15, 29, and 180. The study was approved by the Institutional Review Board of Emory University (9/17/2018).

### 2.2. Assays

#### 2.2.1. Antibody-Secreting Cell ELISpot Assay

The antibody-secreting cell (ASC) ELISpot assay at Day 8 detected antibody-secreting cells against each influenza antigen (from International Reagent Resource: BPL-inactivated A/Michigan/45/2015 FR-1514; A/Singapore/INFIMH-16-0019/2016 FR-1591; B/Phuket/3073/2013 FR-1403; B/Colorado/6/2017 FR-1593) that corresponded with the four strains used in the 2018–2019 FDA-approved influenza vaccine [28]. The influenza antigens were first coated on a plate to provide a capture matrix. Next, one million peripheral blood mononuclear cells (PBMC) from each participant were added to the plate and incubated. Secreted antibodies (specific to H1N1, H3N2, B/Yamagata, and B/Victoria antigens), both IgA and IgG, were captured on the influenza antigen coating and then detected using a biotinylated secondary antibody. A spot-forming substrate was then added to the plates. Each spot formed indicates an antigen-specific ASC, which were manually counted and reported as the numbers of total IgA and IgG ASC per 10^6^ PBMC. 

#### 2.2.2. Hemagglutination Inhibition (HAI) Assay

HAI titers for each of the four influenza strains in the vaccine were obtained at baseline, Day 29, and Day 180 for all participants. In this assay, the reference strains from the quadrivalent 2018–2019 influenza vaccine were obtained from the International Reagent Resource of the Centers for Disease Control (H1N1 FR-1505, H3N2 FR-1590, B Yamagata lineage FR-1364) and the National Institute for Biological Standards and Control (B Victoria lineage 17/254). The HAI assays were performed according to the WHO Influenza Surveillance Network laboratory manual [29] and described previously by our group [30]. Briefly, influenza viruses were propagated in MDCK.2 cells with L-(tosylamido-2-phenyl) ethyl chloromethyl ketone (TPCK) trypsin until cells reached 80% cytopathic effect. Supernatant was collected, and viral titers were determined. Sera were treated with receptor destroying enzyme (RDE) for a final dilution of 1:10. HA titers of H1, H3, and B viruses were determined for RDE-treated and serially diluted sera in 96-well V-bottom plates, mixed with 4 HA units of virus and incubated at room temperature. After incubation, 0.5% turkey RBCs were added and incubated at room temperature. Hemagglutination or inhibition was then recorded for each serum dilution and virus mixture. When there is a sufficient number of anti-HA antibodies, hemagglutination is inhibited and the RBCs precipitate at the bottom of the well (non-agglutinated state). The initial dilution was defined as 1:10 per US Food and Drug Administration recommendations, and sera without any reaction were scored as 5. The plates were read by two independent readers, and the transition point (representing the titer) was visualized and documented.

#### 2.2.3. Statistical Analysis

As a pilot study, the sample size was limited. Analyses include descriptive and graphical summaries. Geometric mean titers with 95% confidence intervals were calculated to compare HAI titers. Mann–Whitney test was used to compare the ASC magnitudes and HAI results for the imprinting groups and the vaccination history groups. Statistical significance was considered at a level alpha = 0.05. Statistical analyses were performed using GraphPad Prism software (Version 8.2.0, La Jolla, CA, USA).

## 3. Results

### 3.1. Demographics

For the H3N2 birth cohort, 70% were male, 50% white, and mean BMI was 25.53. The targeted birth year range for the H3N2 cohort was 1968–1977, with the mean age being 43.3 years (standard deviation 2.3 years). For the H1N1 birth cohort, 40% were male, 60% white, and mean BMI was 25.87. The targeted birth year range for the H1N1 cohort was 1948–1957, with the mean age being 65.5 years (standard deviation 2.1 years). The demographic data are summarized (Table 2).

### 3.2. Imprinting

#### 3.2.1. Antibody Secreting Cells (ASC)

**Figure 1:**
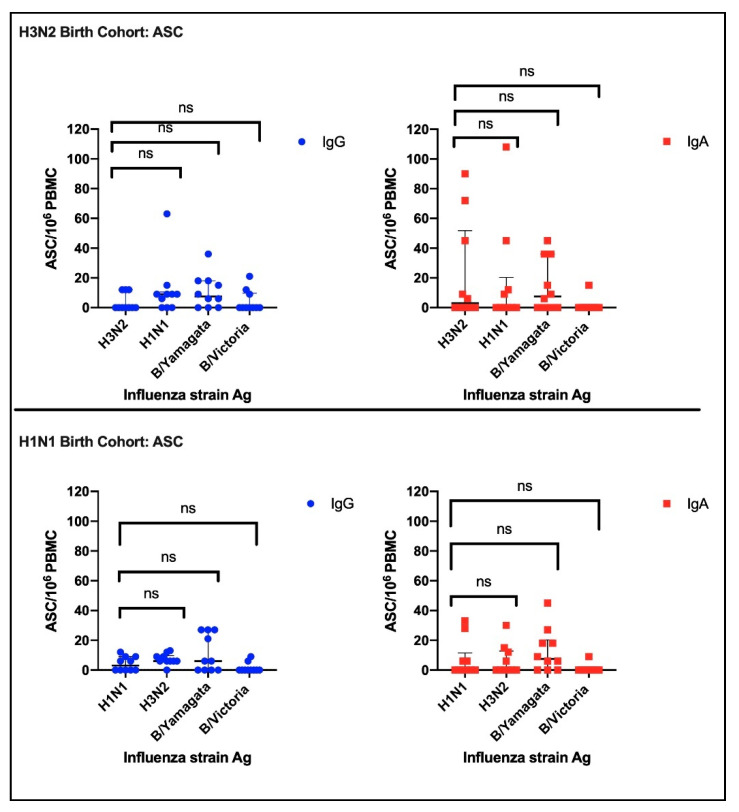
The top panel shows the antibody-secreting cells (ASC) for the H3N2 birth cohort, and the bottom panel shows the ASC for the H1N1 birth cohort. Blue dots are the numbers of antigen-specific IgG-secreting B cells measured for each participant, and red dots are IgA. The black error bars denote median and IQR, and “ns” denotes a non-significant difference.

To assess for imprinting effects, we looked for an ASC dominant response skewed to the subtype circulating around the specified birth years. The numbers of IgG and IgA ASC per 10^6^ PBMC were counted for each influenza antigen contained in the quadrivalent influenza vaccine (Figure 1). For the H3N2 birth cohort, the number of Day 8 ASC detected by the H3N2 antigen was compared independently with either the ASC detected by the H1N1 antigen, B/Yamagata antigen, and B/Victoria antigen (median IgG 9, 0, 7.5, 0 and median IgA 0, 3, 7.5, 0 for H1N1, H3N2, B/Yamagata and B/Victoria antigens, respectively). Similarly, for the H1N1 birth cohort, the ASC detected by the H1N1 antigen was compared independently with the ASC detected by the H3N2 antigen, B/Yamagata antigen, and B/Victoria antigen (median IgG 3, 6, 6, 0 and median IgA 0, 0, 7.5, 0 for H1N1, H3N2, B/Yamagata and B/Victoria antigens, respectively). There were no significant imprinting effects for either IgG or IgA for either birth cohort.

#### 3.2.2. HAI Results

**Figure 2:**
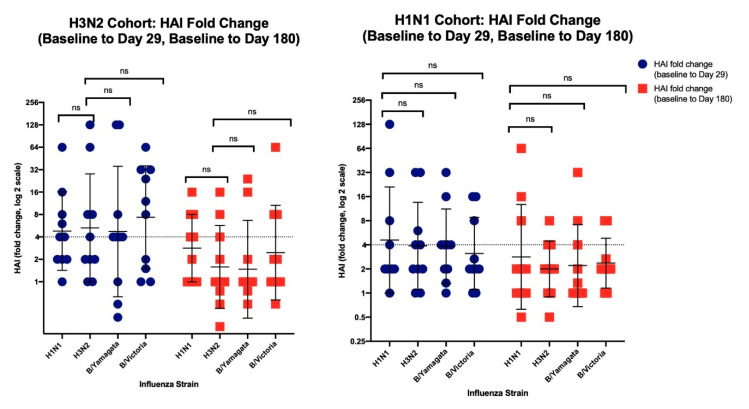
The left panel shows the H3N2 birth cohort and the right panel shows the H1N1 birth cohort. Blue dots are the HAI fold-change from baseline to Day 29, and red squares are the HAI fold-change from baseline to Day 180. The horizontal dotted line at 4 represents the fold change magnitude associated with seroconversion. The error bars denote geometric mean and geometric mean standard deviation, and “ns” denotes a non-significant difference.

The HAI fold-change was documented for each strain at Day 29 and Day 180 (Figure 2). For the H3N2 birth cohort, the HAI fold change for HAI H3N2 titers was compared independently with the HAI fold-change for the H1N1, B/Yamagata, and B/Victoria HAI titers. For the H1N1 birth cohort, the HAI fold-change for the HAI H1N1 titers was compared independently with the HAI fold change for H3N2, B/Yamagata, and B/Victoria HAI titers. For both cohorts, there were no significant differences at either Day 29 or Day 180.

### 3.3. The Effects of Repeated Vaccination

Of the participants recruited for the repeated annual influenza vaccination group, 9/10 subjects had received influenza vaccinations in each of the last five years, and 1 subject had received influenza vaccinations in four of the past five years. In the minimally vaccinated group, 8/10 subjects had received zero vaccinations in the past five years, 1 subject had received 1 vaccination in the past 5 years, and 1 subject had received 2 vaccinations in the past 5 years.

#### 3.3.1. ASC Results

**Figure 3:**
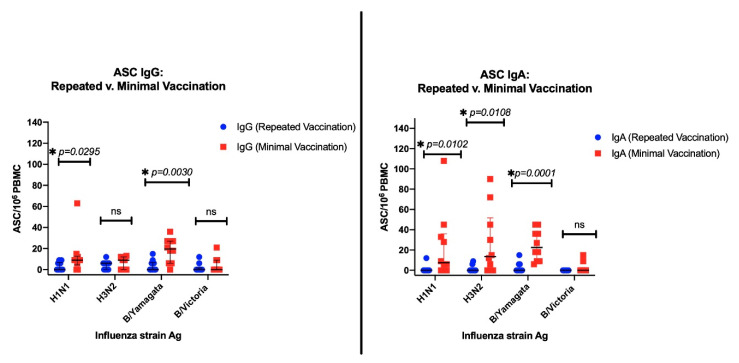
The left panel shows the IgG ASC magnitudes and the right panel shows the IgA ASC magnitudes, with blue dots representing the repeated vaccination group and red squares representing the minimally vaccinated group. The black error bars denote median and IQR. The asterisk (*) denotes a significant difference with *p* < 0.05, and “ns” denotes a non-significant difference.

The antigen-specific IgG ASC were quantified for the repeated versus minimally vaccinated groups, and their numbers were compared for each of the influenza antigens. The group that was minimally vaccinated had a numerically higher IgG ASC response than the repeatedly vaccinated group, and was significant for the H1N1 antigen (*p* = 0.0295) and the B/Yamagata antigen (*p* = 0.0030). Similarly, for the IgA ASC, the minimally vaccinated group had a numerically higher ASC response, which was significant for the H1N1 antigen (*p* = 0.0102), H3N2 antigen (*p* = 0.0108), and B/Yamagata antigen (*p* = 0.0001) (Figure 3).

#### 3.3.2. HAI Results

**Figure 4:**
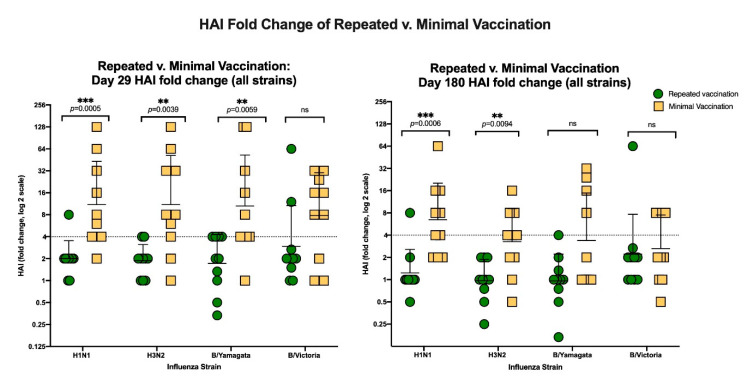
The left panel shows the HAI fold-change from baseline to Day 29 post-vaccination, and the right panel shows the HAI fold-change from baseline to Day 180 post-vaccination. The green dots represent the group that received repeated prior vaccination, and the yellow squares represent the group with minimal prior seasonal vaccination. The horizontal dotted line at 4 represents the fold change magnitude associated with seroconversion. The error bars denote geometric mean and geometric mean standard deviation. The asterisk (**) denotes a significant difference with *p* < 0.01, (***) denotes a significant difference with *p* < 0.001, and “ns” denotes a non-significant difference.

The HAI fold-changes from vaccination to Day 29 and vaccination to Day 180 were measured. The group that had minimal prior vaccinations had a numerically higher HAI fold change than the group that had repeated prior vaccinations, which was statistically significant at Day 29 for the H1N1, H3N2, and B/Yamagata strains (*p* = 0.0005, 0.0039, 0.0059; respectively) and statistically significant at Day 180 for the H1N1 and H3N2 strains (*p* = 0.0006, *p* = 0,0094, respectively; Figure 4).

**Figure 5:**
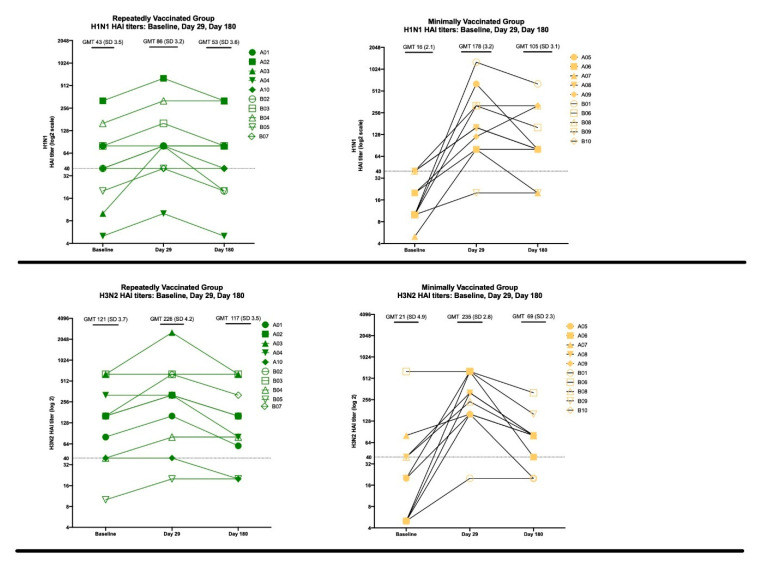

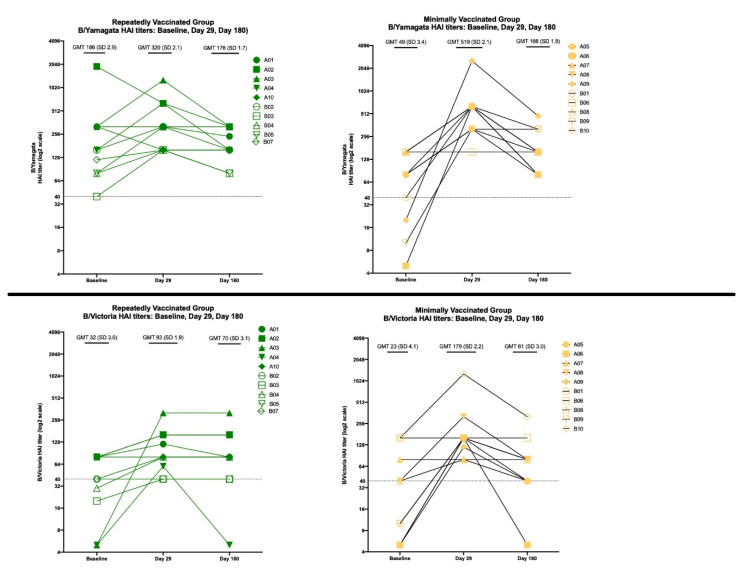
The panels (top to bottom) display the H1N1 HAI, H3N2 HAI, B/Yamagata HAI, and B/Victoria HAI titers for each subject at Baseline, Day 29, and Day 180. Green symbols represent the repeatedly vaccinated subjects, and yellow symbols are the minimally vaccinated group. The closed symbols denote individuals from the H3N2 birth cohort, and the open symbols denote individuals from the H1N1 birth cohort. Geometric mean titer and geometric standard deviation factor are provided. The horizontal dotted lines at 40 represent the HAI titer typically associated with seroprotection.

The geometric mean titers (GMT) were calculated at baseline, Day 29 and Day 180 (Figure 5). The participants in the repeatedly vaccinated group at baseline had numerically higher HAI titers (all ≥ 40, which is considered seroprotective) than the minimally vaccinated group (many < 40). This difference in GMT reached statistical significance for H3N2 HAI baseline GMT (121 v. 21, repeated v. minimal vaccination; *p* = 0.0143) and for B/Yamagata HAI baseline GMT (186 v. 49; *p* = 0.0262), but not for H1N1 (43 v. 16, *p* = 0.0511;) or B/Victoria (32 v. 23; *p* = 0.7186). However, individuals with minimal past influenza vaccinations had a numerically higher GMT at Day 29 (H1N1 86 v. 178, *p* = 0.143; H3N2 226 v. 235, *p* > 0.999; B/Yamagata 320 v. 519, *p* = 0.1418; B/Victoria 93 v. 179; *p* = 0.6310). The titers for this group tended to remain > 40, the level of seroprotection, at Day 180 for all four strains (H1N1, 53 v. 105, *p* = 0.2303; H3N2 117 v. 69, *p* = 0.3468; B/Yamagata 178 v. 166; *p* = 0.7657; B/Victoria 70 v. 61; *p* = 0.6925), though they were not statistically significant.

## 4. Discussion

In this study, the effects of group-level imprinting and influenza vaccination history on the human immune responses were examined by measuring the ASC and HAI titer magnitudes obtained at baseline, followed by one- and six-months post vaccination with the 2018–2019 quadrivalent seasonal influenza vaccine. Although prior studies have used VE and mathematical modeling to analyze birth cohort imprinting effects, with the main outcome measures being protection from influenza infection or influenza disease severity, few studies have described the immune response in humans who have been stratified based on both birth cohort and vaccination history in a prospective manner. Host factors have been described in animal models; however, influenza immunology is much more complex in humans and animal models do not directly translate to the human experience [31,32].

There was no evidence of an imprinting (or birth cohort) effect on ASC or HAI antibody response magnitudes at Day 29 or Day 180. In the subgroup analysis, there were no imprinting effects seen for the birth cohorts even when stratified by vaccination history (Supplemental Figure S1). There are several possible explanations for why our results did not find an imprinting effect. Perhaps despite sharing the same H subtype, the current vaccine viruses have drifted sufficiently from the birth cohort circulating viruses to obscure a demonstrable imprinting effect. It is also possible that the methods used to measure the immune responses in this study—HAI titers and ASC magnitudes—do not fully capture the extent of immune response. For example, although the HAI assay is a standardized measure that serves as a correlate of protection (a titer equal to or greater than 40 is associated with serologic correlate of protection, according to the CDC) [33], it should be acknowledged that a HAI titer ≥ 40 is associated with only 50% clinical protection from infection on a population level. Cellular responses may impact the immune response. Although OAS and imprinting were first described in the context of HA antibodies and the B cell response, there may be a CD4 T cell imprinting impact as well [34]. There may also be a component of neuraminidase (NA) contributing to imprinting effects, or a combination of HA and NA subtype imprinting that defines an individual’s response to influenza infection [8].

Consistent with other reports from the literature, this study found a higher ASC magnitude and higher HAI fold-change in individuals who received minimal vaccinations in the past five years. Individuals in the minimally vaccinated group also tended to maintain an elevated HAI GMT at Day 180 post-vaccination. However, subjects with repeated annual vaccination had higher HAI titers at baseline, before vaccination. These repeatedly vaccinated individuals likely had greater protection at baseline, since their HAI GMTs were mostly greater than 40, whereas the minimally vaccinated subjects mostly had titers less than 40. The “antibody ceiling” effect has been suggested, in which titers do not rise much further if they are already elevated, which has been described in the context of vaccination [35]. For influenza vaccination, several studies have noted a decreased B cell response that is perhaps due to pre-existing serum antibodies due to this ceiling effect [36]. The clinical importance of the antibody ceiling effect (and degree of protection) should be further explored in future studies, especially in regards to repeated annual vaccination and overcoming this effect with other mechanisms (e.g., higher dose or adjuvanted influenza vaccines). The greater magnitude of fold-change response in the minimally vaccinated group was perhaps due to a strong primary response, or to a diminished B cell response in those who have had repeated vaccination. Another mechanism proposed describes reduced serum antibody affinity maturation following vaccination, which has been shown in regards to the H1N1 HA1 domain [37]. In subjects who had received two years of annual influenza vaccination with the same vaccine, the affinities of antibodies in the second year (prior to re-vaccination) were lower than the affinities after the first year of vaccination, which suggests that high-affinity antibodies initially produced by vaccination were not long-lasting. This finding supports prior work that has shown a diminished B-cell response after repeated influenza vaccination, which is perhaps due to activated plasma cells that undergo apoptosis and thus do not produce long lasting effects [38,39].

There are potential limitations of this study. The birth cohorts were defined based on epidemiological data that estimated an individual’s first influenza infection based on his or her birth year. Imprinting effects may not have been seen due to this estimation. For example, for the H1N1 cohort, an individual’s first infection could in fact have been due to H2N2, which was co-circulating in the period around the birth years selected for that cohort. The study design attempted to correct for this effect by targeting individuals born between 1950–1955 (H1N1 birth cohort) and 1970–1975 (H3N2 birth cohort). A separate experiment showed that nine of the ten individuals in the H1N1 birth cohort had baseline HAI titers to a historical H1N1 strain that circulated in the 1950s, which demonstrates that the group was appropriately selected (see Supplemental Figure S2). However, imprinting may occur in response to the first few influenza strains that a child is exposed to, not necessarily the first encounter only. Another important consideration for the birth cohort years regards pandemic years. More people may have been exposed to a specific strain during a pandemic, and therefore the first influenza exposure may not have been to the one identified by birth cohort. The pilot study had only twenty participants, and was intended to be a hypothesis-generating study. As such, the sample size was not based on power calculations.

## 5. Conclusions

In summary, there was no evidence of an imprinting effect. The results showed an increased HAI fold change for individuals who had received minimal influenza vaccination; however, individuals with a history of repeated vaccination tended to have higher and protective titers at baseline. The immune mechanisms relating to repeated vaccination and imprinting will be important to further investigate, in order to develop more effective and durable influenza vaccines. Imprinting and repeated influenza vaccination should not be studied independently, since they likely affect one another. Future studies should analyze immune responses in a larger population, and during different influenza seasons. While our understanding of OAS has deepened since Thomas Francis in 1960, there are likely additional factors, including NA effects and T-cell specific influences that may be contributing to the imprinting effect in humans.

## Figures and Tables

**Table 1:** Components of 2018–2019 Quadrivalent Vaccine.

Influenza A(H1N1)	Influenza A(H3N2)	Influenza B (Victoria Lineage)	Influenza B (Yamagata Lineage)
A/Michigan/45/2015-like virus	A/Singapore/INFIMH-16-0019/2016-like virus	B/Colorado/06/2017-like virus	B/Phuket/3073/2013-like virus

**Table 2:** Demographics.

	H3N2 Birth Cohort, Born between 1968 and 1977 (*n* = 10)	H1N1 Birth Cohort, Born between 1948 and 1957 (*n* = 10)
Mean Age (SD)	43.3 (2.3)	65.5 (2.1)
Mean Birth Year (range)	1974 (1968–1977)	1953 (1949–1955)
Female sex—no. (%)	3 (30)	6 (60)
Minimally Vaccinated—no. (%)	5 (50)	5 (50)
Repeatedly Vaccinated—no. (%)	5 (50)	5 (50)
Race or Ethnic Group—no. (%)		
White	5 (50)	6 (60)
Black or African American	1 (10)	3 (30)
Asian	2 (20)	0 (0)
More than one race	1 (10)	0 (0)
Choose not to report	1 (10)	1 (10)
Hispanic	0 (0)	0 (0)
Mean BMI (SD)	26.53 (4.4)	25.97 (5.2)

## Supplementary Materials

The following are available online at https://www.mdpi.com/2076-393X/8/4/663/s1, Figure S1: Subgroup analysis of HAI fold change (between baseline and 28 days post-vaccination) for each cohort (H3N2 birth cohort, panel A and B; H1N1 birth cohort, panel C and D) by vaccination history. No significant imprinting effects were observed, even when stratified by vaccination history, Figure S2: Baseline HAI titers against the historical H1N1 A/Denver/1957 strain. Left: H3N2 birth cohort. Right: H1N1 birth cohort. 9/10 individuals in the H1N1 birth cohort had titers equal to or greater than 40 at baseline, which demonstrates pre-existing immunity to this strain.
