# Supplementary material for: The Effects of Imprinting and Repeated Seasonal Influenza Vaccination on Adaptive Immunity after Influenza Vaccination
Source: Vaccines (Basel). 2020 Nov 7;8(4):663. doi: 10.3390/vaccines8040663 (PMC7712189; doi:10.3390/vaccines8040663)
Supplement: Supplementary file 1 [file vaccines-08-00663-s001.pdf]

Supplemental Documents

"The Effects of Imprinting and Repeated Seasonal Influenza Vaccination on Adaptive Immunity After Influenza Vaccination." Sherman *et al.*

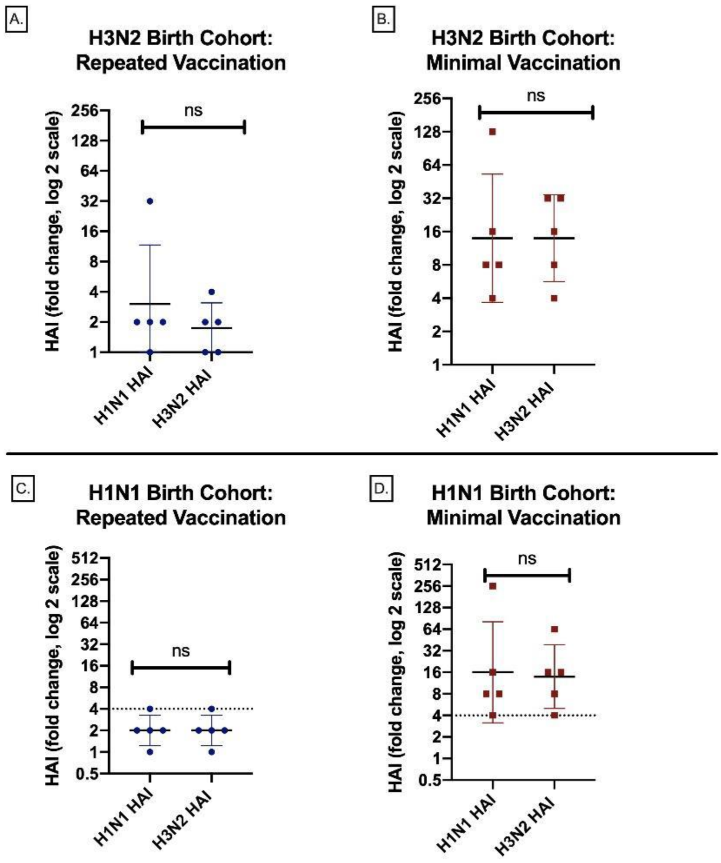

Supplemental Figure 1. Subgroup analysis of HAI fold change (between baseline and 28 days post-vaccination) for each cohort (H3N2 birth cohort, panel A and B; H1N1 birth cohort, panel C and D) by vaccination history. No significant imprinting effects were observed, even when stratified by vaccination history.

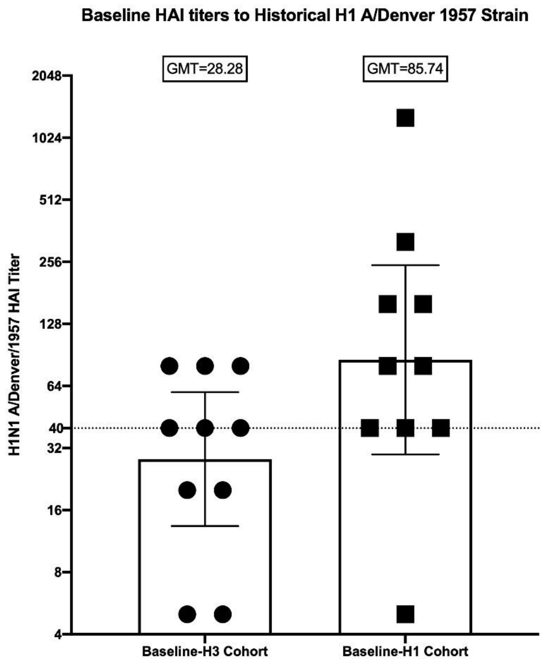

Supplemental Figure 2. Baseline HAI titers against the historical H1N1 A/Denver/1957 strain. Left: H3N2 birth cohort. Right: H1N1 birth cohort. 9/10 individuals in the H1N1 birth cohort had titers equal to or greater than 40 at baseline, which demonstrates pre-existing immunity to this strain.

Supplemental: Inclusion/Exclusion Criteria

Inclusion Criteria:

1. Capable of informed consent and provision of written informed consent before any study procedures.
2. Capable of attending all study visits according to the study schedule.
3. Males or females born between 1968-1977 or 1948-1957.
4. Are in good health, as determined by medical history and targeted physical exam related to this history.
5. Oral temperature is less than 38C.
6. Resting pulse rate is between 50 and 100 beats per minute.
7. Female subjects of childbearing age must have a negative urine pregnancy test within 24 hours before study vaccination.
8. Have received the influenza vaccine at least 3 of the past 5 years (between September 2013-June 2018) or have received the influenza vaccine in 2 or less of the past 5 years (between September 2013-June 2018).

Exclusion Criteria:

1. Have an acute illness within 72 hours before vaccination.
2. Have any condition that, in the opinion of the principal investigator, would place the subject at an unacceptable risk of harm or confound the interpretation of the study results.
3. Have any acute or chronic medical condition that, in the opinion of the principal investigator, would make vaccination unsafe or interfere with the evaluation of immune response to study vaccination.
4. Have a suppressed immune system as a result of illness, immunosuppressive medication, chemotherapy, or radiation therapy within 3 years prior to study vaccination.
5. Have known HIV, hepatitis B, or hepatitis C infection.
6. Have a known history of autoimmune disease.

7. Have taken oral or parenteral corticosteroids of any dose within 30 days before study vaccination.

8. Have taken high-dose inhaled corticosteroids within 30 days before study vaccination.

9. Have received, or plan to receive, any licensed live vaccine within 30 days, or any licensed inactivated vaccine within 14 days, prior to, or after, study vaccination.

10. Have planned receipt of any unlicensed or investigational medications, biologics, or vaccines for the duration of subject study participation.

11. Have received immunoglobulin or other blood products, with the exception of Rho D immunoglobulin, within 90 days prior to study vaccination.

12. Have donated blood or blood products within 30 days before study vaccination, or within 60 days after study vaccination, or plan to donate blood within 30 days of the last blood draw.

13. Have known hypersensitivity or allergy to eggs, egg protein, chicken protein, or other compounds of the study vaccine.

14. Have a history of severe reactions following vaccination with influenza virus vaccines.
